# Biphasic Malignant Pleural Mesothelioma Masquerading as a Primary Skeletal Tumor

**DOI:** 10.1155/2016/7560929

**Published:** 2016-08-31

**Authors:** James Benjamin Gleason, Basheer Tashtoush, Maria Julia Diacovo

**Affiliations:** ^1^Department of Pulmonary and Critical Care Medicine, Cleveland Clinic Florida, 2950 Cleveland Clinic Boulevard, Weston, FL 33331, USA; ^2^Department of Pathology and Laboratory Medicine, Cleveland Clinic Florida, 2950 Cleveland Clinic Boulevard, Weston, FL 33331, USA

## Abstract

Biphasic malignant pleural mesothelioma is a rare malignant tumor, usually presenting as a pleural-based mass in a patient with history of chronic asbestos exposure. We herein report a case of a 41-year-old man who presented with chest pain and had a chest computed tomography (CT) scan suggestive of a primary skeletal tumor originating from the ribs (chondrosarcoma or osteosarcoma), with no history of asbestos exposure. CT-guided core needle biopsies were diagnosed as malignant sarcomatoid mesothelioma. Surgical resection and chest wall reconstruction were performed, confirming the diagnosis and revealing a secondary histologic component (epithelioid), supporting the diagnosis of biphasic malignant mesothelioma.

## 1. Introduction

Mesothelioma is an uncommon and highly aggressive malignant tumor [1] usually presenting as a pleural-based mass in those with history of asbestos exposure. The biphasic (mixed) subtype is the second most common histologic type accounting for 20–35% of all malignant pleural mesotheliomas (MPM). It is characterized by the concomitant presence of epithelioid and sarcomatoid features, the latter associated with worse prognosis [2, 3]. Sarcomatoid and biphasic subtypes of MPM often mimic other malignant and benign conditions on radiographic and histologic examination and, due to their poor prognosis, early diagnosis becomes most imperative [4, 5]. Herein, we report a case of biphasic MPM presenting with chest pain and a rib mass in a healthy middle aged male with no identifiable risk factors.

## 2. Case Presentation

A 41-year-old Caucasian man presented to our institution with left lateral chest pain for approximately one year. He was previously evaluated at an outside institution where he underwent an unrevealing cardiac workup for his chest pain, including cardiac enzyme testing, a treadmill stress test, and echocardiogram. This was followed by a chest CT scan that showed a destructive lesion involving the posterior-lateral aspect of the left seventh and eighth ribs. He had no history of chest wall trauma or injuries. He had no cough, dyspnea, fever, chills, or night sweats. However, he reported a five-pound unintentional weight loss over the last three months. His past medical history was unremarkable, with no history of surgeries, cigarette smoking, or tobacco use and no occupational or environmental exposures. Family history was negative for chronic diseases or malignancies. Physical examination revealed a tender mass along the left lateral chest wall, corresponding to the area where the destructive rib lesion was seen on the previous CT scan.

**Figure 1:**
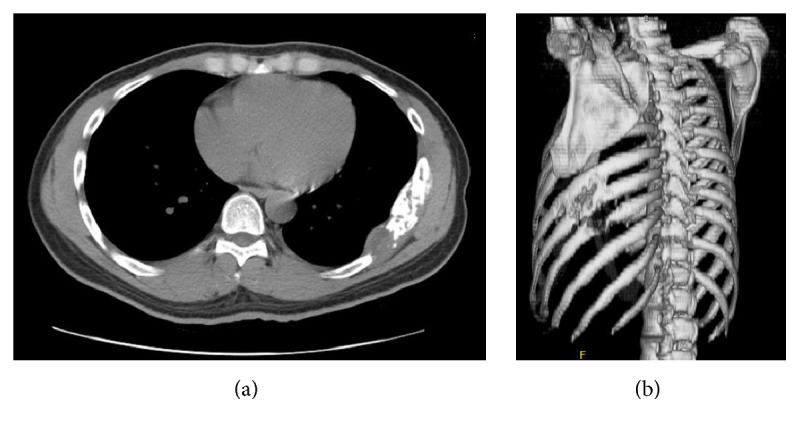
(a) Axial CT chest without contrast and soft tissue views demonstrating the destructive lesion involving the 7-8th ribs. (b) Three-dimensional CT reconstruction of the thoracic cage showing bony destruction of the 7th, 8th, and 9th ribs.

A repeated chest CT, with additional imaging of the abdomen and pelvis, demonstrated an 8.5 × 8.2 × 3.0 cm destructive tumor involving the posterior-lateral aspect of the left seventh, eighth, and ninth ribs (Figure 1), which appeared to primarily involve the bone, with some adjacent soft tissue invasion, and a soft tissue component bulging into the pleural space (Figure 1(a)). There were no other abdominal or pelvic lesions. A primary malignancy of the ribcage such as chondrosarcoma or osteosarcoma was highly suspected. Differential diagnosis also included lymphoma, a plasma cell neoplasm, and Ewing's sarcoma/primitive neuroectodermal tumor (PNET).

**Figure 2:**
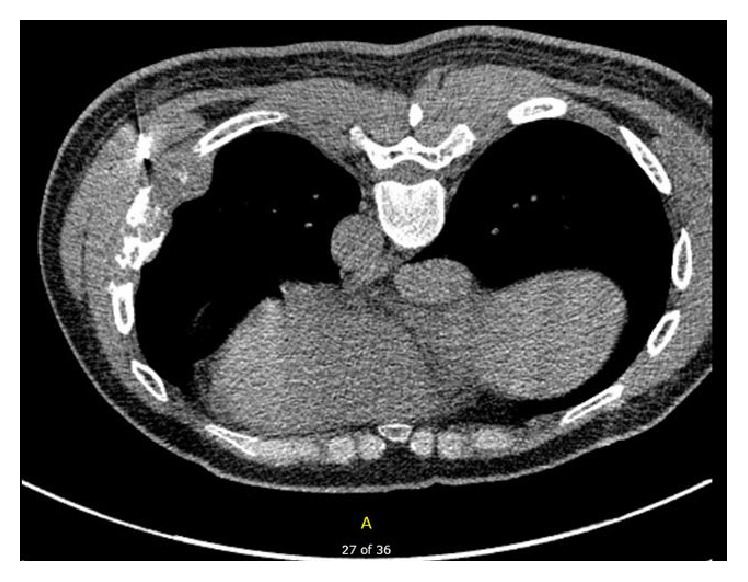
Axial CT chest without contrast, soft tissue views demonstrating the patient in prone position with CT-guided biopsy needle on satisfactory trajectory to the bony and soft tissues lesion.

**Figure 3:**
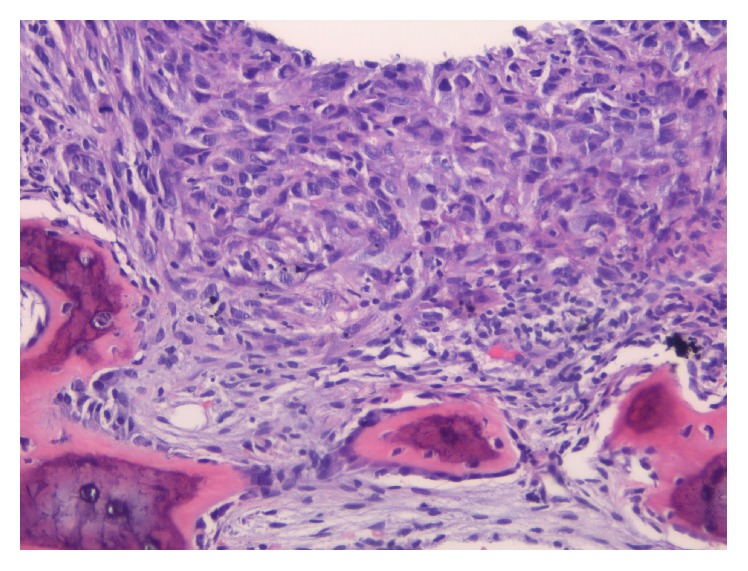
Needle core biopsy, H&E 20x: bone involved by malignant pleomorphic and spindled cells.

A CT-guided core needle biopsy of the rib lesion and adjacent soft tissue was performed (Figure 2). It revealed markedly pleomorphic malignant cells (Figure 3). Morphologically, the differential diagnosis included a sarcoma, a metastatic sarcomatoid carcinoma, melanoma, and sarcomatoid mesothelioma. Immunohistochemistry showed that the malignant cells expressed CAM 5.2 (strong, diffuse), AE1/AE3 (strong, diffuse), D2-40 (patchy, strong), and CK5/6 (patchy, weak) and lacked CK7, CK20, TTF-1, p63, WT-1, calretinin, MOC-31, Ber-Ep4, and S100 reactivity. A sarcomatoid mesothelioma was suspected. An expert second opinion was pursued. The reviewers concurred with the diagnosis based on the morphology, the strong presence of keratins (CAM 5.2, AE1/AE4), expression of D2-40 (patchy but strong), and absence of other epithelial/melanoma markers indicating a metastatic process.

**Figure 4:**
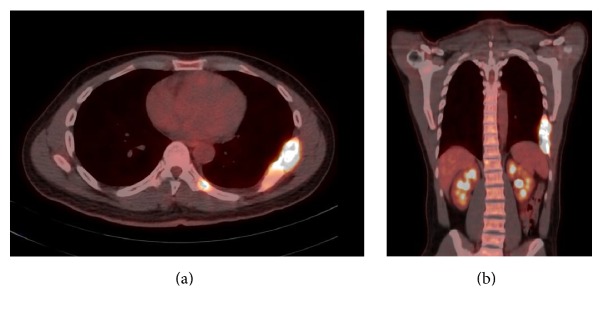
(a) Axial PET-CT demonstrating avid FDG uptake in the destructive lesions involving the 7th, 8th, and 9th ribs. Also a notable focus of FDG uptake is seen left of the T12 vertebra. (b) Coronal PET-CT demonstrating avid FDG uptake in the destructive lesion involving the 7th, 8th, and 9th ribs.

A positron emission tomography- (PET-) CT scan was performed for staging and showed FDG uptake within the destructive chest wall lesion with standardized uptake value (SUV) of 17, an additional area of FDG uptake was also seen on the left side of the twelfth thoracic vertebra (T12) with SUV of 4 (Figure 4).

Treatment with combination pemetrexed-cisplatin chemotherapy was initiated, with a total of 4 cycles. A follow-up chest CT showed a stable 8 cm rib-pleural mass. After presenting and discussing the case at a multidisciplinary thoracic oncology conference, consensus was in favor of surgical resection with chest wall reconstruction.

**Figure 5:**
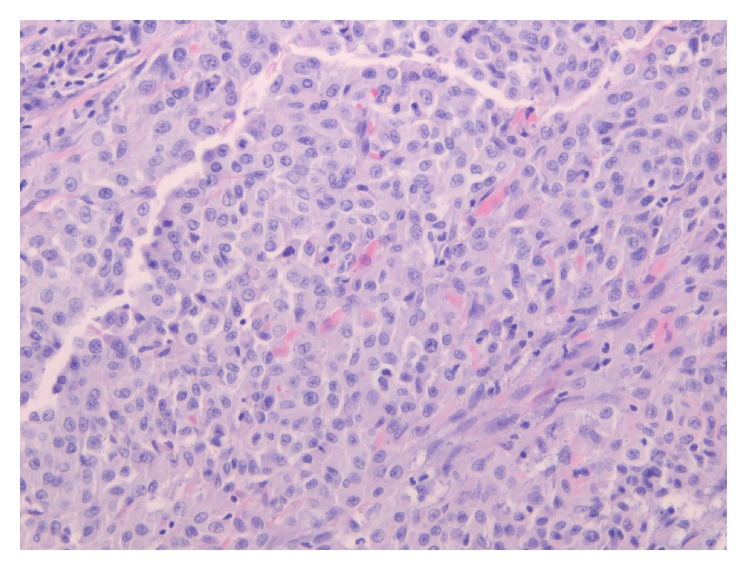
Resection specimen, chest wall tumor, H&E 20x: rare epithelioid foci present in the 10 cm mass and involving the lung parenchyma.

**Figure 6:**
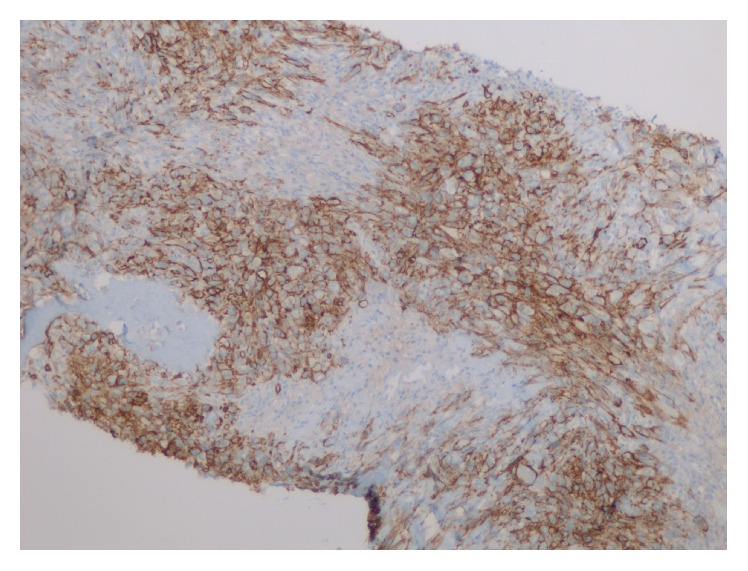
Resection specimen, chest wall tumor, H&E 5x: the epithelioid component is evident in the invaded lung parenchyma.

**Figure 7:**
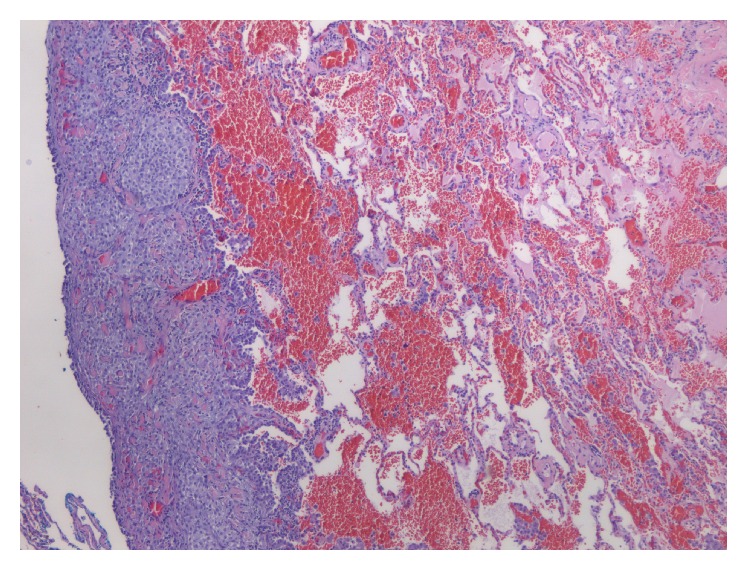
Resection specimen and chest wall tumor: D2-40 IHC-positive foci, sarcomatoid component.

One month later, the patient underwent a left thoracotomy procedure with tumor resection, left upper and lower lobe pulmonary wedge resections, and chest wall reconstruction with titanium mesh plate and latissimus dorsi flap. Surgical gross pathology showed a large, rib-based 8 cm tumor and two lung parenchymal wedge biopsies with white-tan pleural-based nodules. Interestingly, while the tumor revealed histological features similar to the previous biopsy, the lung specimens showed involvement by a mesothelioma with epithelioid features (Figures 5 and 6). Immunohistochemical analysis demonstrated D2-40 positive foci within the sarcomatoid component (Figure 7) similar to the core needle specimens. The combination of both histologic components warranted the diagnosis of biphasic MPM. The tumor was staged as T4N0M1 (stage IV).

**Figure 8:**
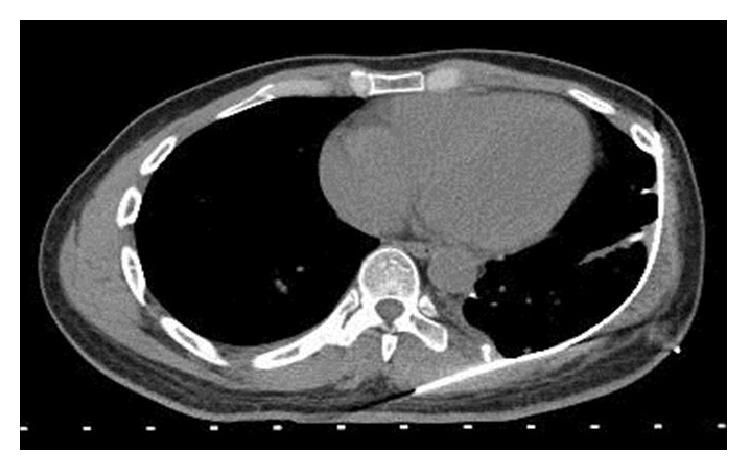
Follow-up axial CT chest without contrast demonstrating removal of pleural soft tissue mass, skeletal lesions, and titanium mesh chest wall reconstruction.

After allowing time for the surgical recovery and wound healing, the patient was referred to radiation oncology, where he received left chest wall radiation for positive resection margins. Follow-up CT imaging has not shown recurrence after ten months of follow-up (Figure 8).

## 3. Discussion

MPM is a highly aggressive cancer typically associated with asbestos exposure [6–8]. Patients typically present with chest pain and a pleural effusion that may conceal an underlying pleural mass. It usually spreads by local invasion, often involving the chest wall and/or diaphragm. MPM is histologically subclassified as epithelioid, biphasic, or sarcomatoid. The epithelioid type is often easier to identify on histopathology and carries a better prognosis among all. Biphasic tumors are defined by the presence of a combination of the epithelioid and sarcomatoid components in close proximity, and prognosis is often dependent on the predominant type [9]. However, biopsy specimens suggesting MPM with sarcomatoid features may still need to be differentiated from other types of malignancies, since they may express a paucity of immunohistochemical markers.

Based on the postmortem analysis of 172 patients with MPM, most commonly involved distant organs include the liver (55.9%), adrenal glands (31.3%), kidneys (30.1%), and contralateral lung (26.8%) [10]. Exceedingly rare presentations are those with breast and intracranial metastases [11–13]. MPM has 4 : 1 predominance in males [14] and the mean age of diagnosis is 60 years. It has a very strong relationship with asbestos exposure, reported in up to 80% of cases [15].

This case of biphasic MPM is unique and challenging, as it affected a young patient, with no history asbestos exposure, and exhibited an unusual presentation, suggesting a primary skeletal malignancy. Furthermore, it succinctly highlights limitations and possible clinical pitfalls in the diagnosis of MPM. As with other tumors, the larger the lesion is, the less representative a core biopsy or fine needle aspiration (FNA) may be. In addition, if a tumor is poorly differentiated (i.e., sarcomatoid), loss of phenotypic marker expression is not uncommon [16, 17]. Kao et al. demonstrated this sampling limitation and frequent misclassification of nonepithelioid mesotheliomas and concluded that adequate surgical biopsies increased the accuracy over radiology-guided needle core/FNA biopsies [18].

MPM is highly resistant to therapy and the preferred treatments are surgery (at early stages) and/or combination treatments with radiotherapy and chemotherapy [19]. Conventional therapy with cisplatin and pemetrexed allows only palliation for the majority of patients and the average survival time after diagnosis remains poor [20–22]. How well a biphasic tumor responds to treatment depends on the ratio of epithelial to sarcomatoid cells. A tumor composed mostly of epithelioid cells tends to grow slower and responds better to treatment [23, 24].

## 4. Conclusion

When evaluating suspected skeletal tumors based on thoracic imaging, variants of malignant pleural mesotheliomas must always be considered in the differential diagnosis of a chest wall lesion, even in relatively young individuals with no history of asbestos exposure. The identification of nonepithelioid subtypes of malignant pleural mesotheliomas may require extensive workup, exhausting the limited material in core/FNA biopsies. In addition, they may not be entirely representative of all the histologic components present in the tumor. Nonetheless, these procedures remain invaluable as the first step in guiding further diagnostic and therapeutic interventions.
